# The Potential of Bioelectrochemical Sensor for Monitoring of Acetate During Anaerobic Digestion: Focusing on Novel Reactor Design

**DOI:** 10.3389/fmicb.2018.03357

**Published:** 2019-01-15

**Authors:** Hao Sun, Irini Angelidaki, Shubiao Wu, Renjie Dong, Yifeng Zhang

**Affiliations:** ^1^Key Laboratory for Clean Renewable Energy Utilization Technology, Ministry of Agriculture, College of Engineering, China Agricultural University, Beijing, China; ^2^Department of Environmental Engineering, Technical University of Denmark, Lyngby, Denmark; ^3^Aarhus Institute of Advanced Studies, Aarhus University, Aarhus, Denmark

**Keywords:** bioelectrochemical system, biosensor, acetate, sensitivity, anaerobic digestion

## Abstract

Acetate as the dominant fraction of volatile fatty acids (VFAs) is an important intermediate in metabolic pathways of methanogenesis, which could reflect the stability status of anaerobic digestion (AD) process. Bioelectrochemical sensors for environmental or bioprocess monitoring have become increasingly attractive in recent years. Although it was more favorable, several challenges still need to be addressed for acetate detection, including large electrode spacing, low stability, biofouling at the cathode and low detection range. In this study, an innovative biosensor on the basis of a three-chamber microbial electrochemical system was proposed to monitor the acetate during the AD process. In such a system, acetate was first transferred from sample chamber through the anion exchange membrane (AEM) to anode due to the driven force of concentration difference and then oxidized by anodic biofilm as a substrate for the current generation. With such design, the influence of waste properties fluctuation in the cathodic reaction could be avoided. The response of current density to different acetate concentrations was investigated. The selectivity, the influence of the sample temperature and the external resistance were also evaluated. The correlation (*R*^2^ > 0.99) between the current densities and acetate concentrations (up to 160 mM) was established at specific reaction time (from 2 to 5 h). Current densities after 5 h reaction were improving about 20% when the sample temperature was high (e.g., 37 and 55°C). The detection range increased along with the decrease of external resistance. The acetate concentrations of AD effluents as determined by the biosensor where within 24.2% of the ones determined by gas chromatography. Nevertheless, the application of the biosensor for monitoring acetate in environmental samples could still be promising.

## Introduction

The application of microbial electrochemical technologies as biosensors for environmental or bioprocess monitoring has attracted considerable research interest in recent years (Zhou et al., 2017; ElMekawy et al., 2018). These biosensors rely on electroactive biofilms that is capable of transferring the electrons extracted from the metabolic oxidation of organic substrates to an electrode (Bond et al., 2002). The analytical signal is from the current generated by consuming organic compounds, which directly depends on the microbial activity and the electron donor availability (Prévoteau and Rabaey, 2017). Based on an archetype, microbial fuel cell (MFC), multiple biosensors were proposed for water quality measurement with this concept (e.g., biochemical oxygen demand and toxic components; Abrevaya et al., 2015a,b; ElMekawy et al., 2018). Among which, one promising and important application is the detection of volatile fatty acids (VFAs) during anaerobic digestion (AD) or fermentation processes (Atci et al., 2016; Jin et al., 2017; Kretzschmar et al., 2017).

The stability and efficiency of the AD process are dependent on the concerted and syntrophic activity of microorganisms which are sensitive to variations of the operating conditions such as temperature, organic loading amount and composition (Vanwonterghem et al., 2014). VFAs are important intermediates in metabolic pathways of fermentation and methanogenesis, which also has been proven to be a reliable stability indicator that sensitive to the system stress. Thus, the accumulation of VFAs could reflect a kinetics unbalance between its producers and consumers in AD possess (Ahring et al., 1995). Especially in recent years, in the light of “power on demand”-concept, a high flexible feeding strategy for biogas production is necessary, which causes an urgent need for an effective monitoring in order to achieve an optimal AD process control (Hahn et al., 2014). However, conventional methods for VFAs monitoring are usually expensive, time-consuming, mostly offline and require extensive laboratory work, such as titration, liquid or gas chromatography (Sun et al., 2017).

In this context, bioelectrochemical biosensors such as MFC biosensor has been considered by several investigators and it could become a promising and convenient solution for the online, *in situ* monitoring of the VFAs with sufficient accuracy (Kaur et al., 2013; Chouler and Di Lorenzo, 2015). Though it has obvious advantages, development of such MFC based biosensor for acetate monitoring is still challenging. Separation of the sample and anodic biofilm in two compartments by an anion exchange membrane (AEM) was proved to be an effective solution for selectively monitoring VFAs rather than other organic compounds typically presented in AD slurry (Jin et al., 2016, 2017). However, adding a middle chamber between anode and cathode for receiving digestate may increase the electrode spacing, thereby increasing internal resistance (Jin et al., 2016). Receiving digestate in cathode chamber could solve this issue but the growth of aerobic bacteria on the surface of cathode electrode may deteriorate the sensor stability during long-term operation. Another key challenge of the aforementioned system is that the detection range is still narrow. The main driving forces in these bioelectrochemical sensors are ionic migration driven by the potential difference and concentration gradient between the anode and cathode. Since the dependence of current generation by anodic bacteria to substrate concentration is following the Monod equation, lowering the faction of VFAs transferred from the sample chamber to the anode could broaden the detection range.

**FIGURE 1:**
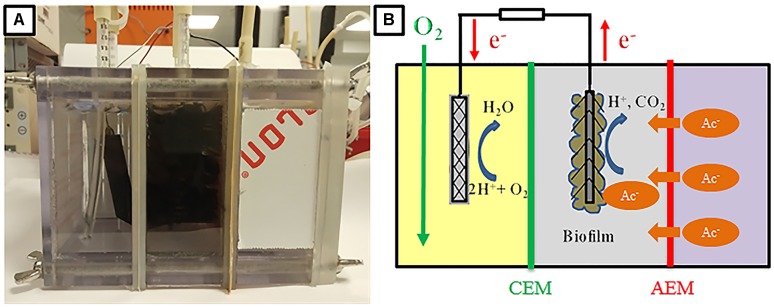
Prototype **(A)** and schematic diagram **(B)** of the MFC based biosensor.

Therefore, in the present study, we developed an innovative three-chambered bioelectrochemical sensor for acetate detection. The novel biosensor was composed of sample chamber, anode chamber and cathode chamber, which are separated by (AEM) and cation exchange membrane (CEM) separating the sample-anode chamber and anode-cathode chamber, respectively (Figure 1). In the biosensor, due to the concentration gradient, acetate ion in digestate would be first transported from the sample chamber through the AEM to anode chamber where it could be oxidized by electroactive bacteria for the current generation. Instead of locating the sample chamber in the middle, the present sensor design has the advantage of maintaining a short electrode spacing. Since the concentration gradient is the only driving force for acetate transportation, the faction of acetate transferred from digestate to anode could be decreased, thereby broadening the detection range. To the best of our knowledge, such bioelectrochemical sensor design has never been reported for acetate detection. To demonstrate the feasibility and obtain a better understanding of the performance of the biosensor for the application in AD process monitoring, several experiment setups were conducted and described as follows. Firstly, the response of current densities in the biosensor under various acetate concentrations with artificial wastewater (simulating AD effluent) was tested and the acetate transportation mechanism was also investigated. Subsequently, the cross-influence of other VFA present in samples such as propionate and butyrate was evaluated. Furthermore, the influences of the sample temperature and external resistance on the performance of biosensor were studied. Finally, the reliability of the biosensor was verified by testing the effluent from a continuous stirring tank reactor (CSTR) fed with cow manure. The outcomes of this study would benefit the AD process monitoring and optimization and also expand the application of microbial electrochemical technologies.

## Materials and Methods

### Biosensor Setup and Operation

The three-chamber bio-electrochemical reactor used in the present study was constructed by adding one sample chamber beside the anode chamber of a traditional two-chamber MFC reactor, as shown in Figure 1. Chambers (sample, anode, and cathode) with the same dimensions (8 cm × 8 cm × 4 cm) were physically separated by an AEM (AMI 7001, Membranes International, NJ, United States, 9 cm × 9 cm) and a CEM (CMI 7000, Membranes International, NJ, United States, 9 cm × 9 cm). These rectangular compartments were made of non-conductive polycarbonate plates, which possessed three ports on the top for electrodes, and rubber tubes were inserted for medium refilling, aeration, and sampling. Stainless screws and rubber gaskets were used to assemble the reactor and prevent leakage. Carbon fiber brush (5.0 cm in diameter, 7.0 cm in length, Mill-Rose, United States) that had been heat-treated in a muffle furnace at 450°C for 30 min was used as anode electrode (Feng et al., 2010). The cathode electrode was made of graphite plate (5.2 cm × 5.2 cm) coated with 0.5 mg/cm^2^ platinum. Both the electrodes were connected to an external resistance (10 Ω).

The effluent of the anode in an MFC which contained an established biofilm (inoculated by domestic wastewater from primary clarifier and enriched with the acetate as sole substrate) was used as the inoculum to form the exoelectrogenic biofilm on the anode electrode. The cultivation medium for the biofilm was a 50 mM phosphate buffer solution supplemented with trace elements and vitamins (Na_2_HPO_4_, 4.33 g/L, NaH_2_PO_4_, 2.03 g/L, NH_4_Cl, 0.31 g/L; KCl, 0.13 g/L; 12.5 mL mineral solution and 12.5 mL vitamin solution) as previously described (Jin et al., 2017). Acetate (15 mM) was the sole substrate. The cathode chamber was filled with 250 mL of 50 mM phosphate buffer (pH: 7.33 ± 0.06, EC: 6.64 ± 0.04) and continuously aerated with around 300 mL/min flow rate. The sample chamber was filled with deionized water except under the experimental conditions. During the biofilm enrichment period, the solution in three chambers was replaced with new when the voltage across the 1000 Ω external resistance was lower than 50 mV. After the solution was renewed several times, the mature electrochemically active biofilm formed with the system voltage stabilizing at around 650 mV.

The resistance was changed from 1000 to 10 Ω after enrichment and cultivation period, since 10 Ω resistance was adopted in most experiment sets of current work, 1 week adaptation conducted before the experiments. All experiments were conducted in batch mode, and each lasted for 5 h at room temperature (22 ± 2°C). In each batch experiment, the anode chamber was filled with approximately 250 mL buffer solution (pH: 7.31 ± 0.05, EC: 6.64 ± 0.04) containing 50 mM phosphate buffer and nutrient solution (Jin et al., 2017), and purged with N_2_ in 10 min to maintain the anaerobic environment. The anode chamber was flushed with oxygen-free deionized water before refilled with its medium. The solutions in both the anode and cathode chamber were renewed before each batch experiment, and a waiting time for about 2 h was applied to decreasing the voltage to maintain the same initial conditions. Usage synthetic wastewater was based on the modified anaerobic (BA) medium (Angelidaki et al., 1990) (omitted the stock solutions C and E) with a supplement of varying concentrations of analyte. The experiment was conducted in duplicate with two identical reactors.

### Electrochemical Analyses and Calculations

The voltage across the external resistance was monitored using a virtual instrument based data logging system (National Instruments, LabVIEW 2012), connecting to an analog input on the I/O card (USB 3252, ZLAD, China). The voltage signal was acquired every second and average voltage per minute was recorded. The current (*I*) was calculated according to Ohm’s law. The current density (*i*) was calculated as follows:

i=I/A

where *I* (A) is the current and *A* (m^2^) is the projected surface area of the cathode electrode.

The conductivity and pH of the solution in three chambers were measured at the start and the end of each batch using a CDM 83 conductivity meter (Radiometer) and a PHM 210 pH meter (Radiometer), respectively. VFAs concentrations were measured using a GC with FID detection (Thermo Fisher Scientific, TRACE1300).

## Results and Discussion

### Response of Current Density to Various Acetate Concentrations

**FIGURE 2:**
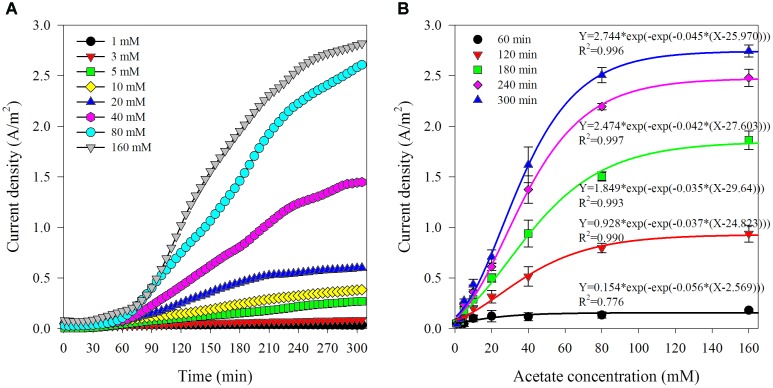
Typical variation of current density corresponding to different acetate concentration in the artificial AD effluent **(A)** and the relationship between current density and acetate concentrations at varied reaction time (from 1 to 5 h) **(B)**.

The first set of experiments was to investigate the response of this biosensor to varied acetate concentrations and establish the calibration. The sample chamber was filled with 250 mL synthetic wastewater contained acetate (1–160 mM). Samples were retrieved from anode effluent for VFAs analysis at the end of each batch. A typical time course of current density corresponding to different initial acetate concentrations is shown in Figure 2A. In general, after a lag phase for about 0.5–1 h, the current density kept increasing along with time. As the initial acetate concentration rose, the current density also increased and showed an *S*-type growing trend. It was observed that the increase in current density turned to be limited when acetate concentration exceeded 80 mM, which indicated that the biosensor was approaching to saturated state due to the rising acetate compared with the limited oxidation capability of biofilm in the anode chamber (Liu et al., 2011). Since there were not any organics in the anode chamber at the beginning of batch run, the increase of current density could be the result of acetate accumulation in the anode chamber. It has been previously reported that the acetate consumption rate was slower compared with its transportation speed through AEM (Jin et al., 2016). It could be assumed that acetate transfer rate through AEM would be increased with the higher concentration gradient between the sample and anode chamber (Kim et al., 2007).

The current densities observed under various initial acetate concentrations at specific reaction time (at 1, 2, 3, 4, and 5 h) are shown in Figure 2B. The relationship between the current densities and the initial acetate concentrations was non-linear regardless of test reaction times, which can be fitted by using a sigmoidal growth model termed Gompertz. The function is shown as follows:

(1) $$i=a*e^{-e(-k*(X-x_{c}))}$$

where, *i* (A/m^2^) is the current density; *X* (mM) is the initial acetate concentration; *a* is amplitude; *x_c_* is center; and *k* is coefficient.

The correlation coefficient factors (*R*^2^) of the non-linear fitting are more than 0.99, except 0.78 for the current densities generated at 1 h. The sigmoidal response from this biosensor is coinciding to the commonly followed *S*-shaped bacterial growth. This is also in line with the reports that non-linear response of current generation by electroactive biofilm to the increase of acetate concentration from 0 to 3 mM in a CSTR type vessel (Kretzschmar et al., 2017). The lowest correlation coefficient factor of the non-linear fitting for the current densities obtained at 1 h indicated that 1 h reaction time was not enough to acquire relatively satisfactory results. The possible reasons could be as follows. Firstly, it took time for acetate to diffuse from sample chamber to anode chamber due to the low mass transfer coefficients and diffusivities for acetate through AEM (5.5 × 10^∧^–8 cm/s, 2.6 × 10^∧^–9 cm^2^/s) (Kim et al., 2007). Secondly, the non-stirring condition was delaying effective mass transfer, and consequently resulting in a short delay of acetate consumption by electrogenic bacteria in the anode chamber. Thirdly, the early stage of the batch experiment (especially the first hour) would be more vulnerable to being affected by the initial conditions which were not exactly the same between batches. For instance, a slight difference in the initial current densities ranging from 0.004 to 0.091 A/m^2^ was observed. Therefore, reaction duration of 1 h reaction time was inadequate to obtain a reliable result. Moreover, the average relative standard deviations of the current densities from the biosensor with various acetate concentrations (1–160 mM) at specific reaction time (2, 3, 4, and 5 h) were 22.10 ± 10.26%, 14.33 ± 7.90%, 9.57 ± 5.68%, and 8.71 ± 4.71%, respectively. This implied that a reasonable accuracy of the biosensor could be obtained within reaction time duration of a few hours. The minimum relative standard deviation was obtained at the longest reaction time (5 h), which implied that long reaction time could improve the biosensor accuracy. Comprehensive consideration of reaction time and accuracy are necessary for the practical application of biosensor. Five hours measuring time would be adequate for acetate monitoring in the AD processes which often have hydraulic retention times in the range of several days (Jin et al., 2016).

A linearity response is also pursued from the aspect as a sensor. As shown in Supplementary Figure S1, linear relationship between the current density and acetate concentration was existing at lower initial acetate concentrations (<40 mM) at specific reaction time (2, 3, 4, and 5 h) with the correlation coefficient factors (*R*^2^) all around 0.99. The average relative standard deviations of current densities at the specific reaction time were 27.01 ± 6.55%, 17.77 ± 5.95%, 11.96 ± 4.44%, and 10.75 ± 3.59%, respectively. The longer reaction time and the steeper slope of the linear fitting model was obtained. This linear response is in line with the previous report that current density increases linearly with VFAs level is from 1 to 30 mM in a three-chamber MDC biosensor for VFAs monitoring (Jin et al., 2016). During this stage, the acetate diffused through AEM and accumulated in anode chamber would maintain low level and limit the increase of current density. The linear correlation between the variation of current density from a biosensor and acetate concentration slowly increasing from 0 to 0.5 mM in the flow cell setup has also been reported (Kretzschmar et al., 2016). The high initial acetate concentration would promote the diffusion and also quicken the acetate accumulation in the anode chamber, which would then reducing the reaction time for approaching to the saturated state in the bioreactor and result in the subsequent non-linear response. Since a wider detection range of more than 40 mM is important for practical application where the effluent from an AD process is under stress condition containing sufficient acetate concentrations (Rajagopal et al., 2013). Hence, the detection result obtained by solving the non-linear fitting equation (Equation 1) is recommended.

(2) $$X=x_{c}-\ln[-\ln(i/a)]/k$$

where, *X* (mM) is the acetate concentration in sample; *i* (A/m^2^) is the current density; *a* is amplitude; *x_c_* is center; and *k* is coefficient.

Overall, the above results demonstrate the feasibility of the biosensor for acetate monitoring in the range of 1–160 mM with a reaction time of more than 2 h.

### Mechanism of Acetate Transportation

**FIGURE 3:**
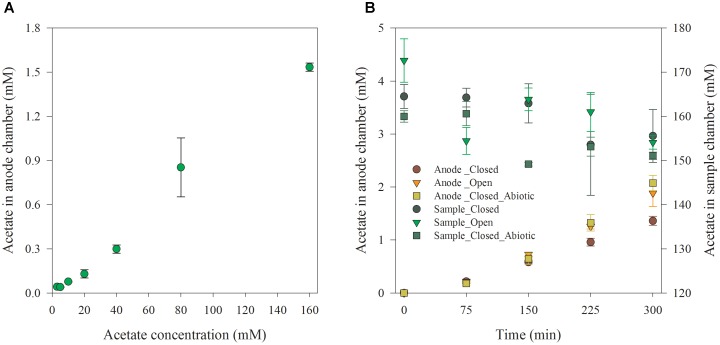
Acetate accumulation in anode chamber at a reaction time of 5 h **(A)** and acetate variation under open and closed circuit conditions along with reaction time **(B)**.

Afterward, the diffusion performance of the acetate from the sample chamber to anode chamber was tested. The sample chamber was filled with synthetic wastewater containing 160 mM acetate. The variation of acetate in sample and anode chamber under the closed and open circuit condition was monitored at an interval of 75 min. Control experiments without microorganisms at anode was also conducted to check the migration of VFAs under such conditions. The accumulated acetate concentration in the anode chamber at the end of the batch runs under different initial acetate levels is shown in Figure 3A. Obviously, the accumulated acetate concentration in the anode chamber increased with the initial acetate level in artificial wastewater that was dosed in sample chamber at the beginning of each batch. Theoretically, in current biosensor design, a concentration-dependent diffusion could be the only mechanism responsible for acetate transportation from the sample to the anode chamber. The diffusion rate through AEM would be proportional to the acetate concentration difference across the AEM, since the membrane area and thickness, and diffusion coefficient were fixed (Kim et al., 2007).

Furthermore, the time-course of the acetate accumulation in the anode chamber and acetate concentration decrease in the sample chamber with an initial acetate concentration of 160 mM under open and closed circuit conditions are shown in Figure 3B. As expected, the acetate concentration in anode chamber increased along with the testing time. Meanwhile, it decreased in the sample chamber. The accumulated acetate concentration in anode chamber under the closed circuit condition was 27.76% lower than that under open circuit condition after 5-h of operation. This was consistent with the report that the accumulated VFAs in the anode under closed circuit operation was lower than that under open circuit or abiotic condition in a MEC-based biosensor due to VFAs’ consumption by anodic exoelectrogenic biofilm for electricity generation (Jin et al., 2017). Moreover, the acetate accumulation under the closed circuit condition was a bit lower than the abiotic condition (closed circuit). A very small amount of acetate could be consumed for maintaining the potential difference (820 mV) between two electrodes when the circuit was open. In addition, it was noticed that the accumulated VFAs concentration in the anode under closed circuit was almost equal to that under open circuit condition in the first 75 min. This agrees with the lag phase and low level of current density at the beginning of the batch which is due to the lag phase for initiation of bacterial acetate consumption.

### The Selectivity of the Biosensor

It has been demonstrated that a wide range of soluble or dissolved complex organic matters can be utilized for electricity generation in MFCs (Pandey et al., 2016). AD effluent usually contains VFAs and other organic matters (such as sugars, lipids, and proteins) that can serve as an alternative substrate for the electroactive biofilm and may affect the sensor selectivity. Thus, it is particularly interesting to investigate the effect of these interfering components on the biosensor performance for a better understanding on the selectivity of this biosensor (Kretzschmar et al., 2017). Since the AEM was used to separate the sample and the anode chamber in the biosensor and block the transportation of non-ionic complex organic matters (such as glucose, lipid, and protein), their interference in acetate monitoring can be avoided (Jin et al., 2016, 2017). However, other VFAs could also transfer along with acetate to anode and overestimate acetate detection, a point only superficially addressed in previous studies. The biofilm in this study was inoculated with the effluent from anode chamber of an MFC which used the acetate as the sole substrate. Thus, the biofilm was supposed to adapt to acetate (Liu et al., 2008).

**FIGURE 4:**
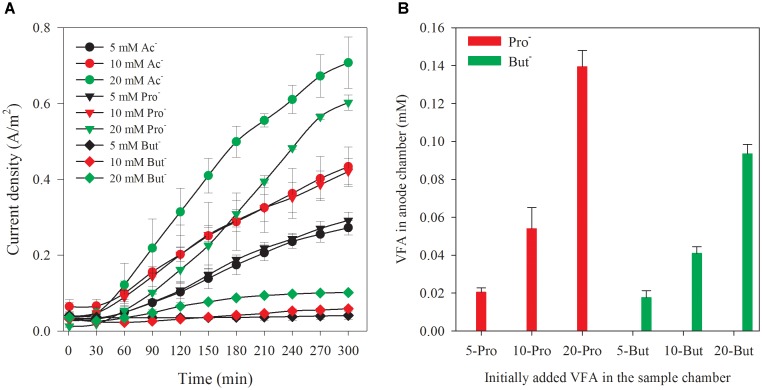
Current density variation along with time corresponding to different VFAs **(A)** and accumulated VFAs at a reaction time of 5 h in anode chamber with different initially added VFAs in the sample chamber **(B)**.

For elucidating the influence of other VFA on acetate accuracy measurements of the biosensor, synthetic wastewater containing sole analyte (acetate, propionate, and butyrate) at three different concentrations (5, 10, and 20 mM, respectively) was tested. The response of current densities to different VFAs (with the concentrations of 5, 10, and 20 mM) is shown in Figure 4A. The current densities increased with the increase of reaction time, and initial concentration of VFAs filled into the sample chamber. The current density generated from propionate approached to the level of that generated from acetate in the same concentration. Comparatively, the current density generated from butyrate was much lower compared with acetate and propionate. The current density generated at 5, 10, and 20 mM propionate was 107.1, 97.0, and 85.2% of the value obtained from acetate, respectively. For the biosensor filled with butyrate, the current densities were only 14.0, 14.0, and 16.8% of that from the acetate (5, 10, and 20 mM), respectively. The accumulation of the propionate and butyrate in anode chamber at the end of the batch is shown in Figure 4B. Obviously, they increased with the rise of initial concentration added in the sample chamber. The increase of current density along with the reaction time demonstrated that all these VFAs were utilized for electricity generation in the anode chamber. It is well accepted that acetate is the most preferential substrate for electricity generation in MFC rectors resulting in the highest coulomb efficiency (CE) (Chae et al., 2009). In addition, the propionate and butyrate are known to be difficult to be degraded in bioprocesses due to their less favorable thermodynamic properties (Kaur et al., 2013). However, the electric current generated from propionate and butyrate was much higher than that we expected in this study. The interference from other VFAs in the acetate measurement with this biosensor cannot be ignored, especially propionate. This possibly due to the facts the acclimated anodic bacterial consortia did not have a high selectivity toward acetate. Thus, this biosensor can be used for acetate measurement only when acetate is the sole or at least the dominant organic acid. More efforts are needed for development of biosensor for acetate measurement. Selecting microbial communities or genetic engineering to regulate specific metabolic pathways of anodic bacteria could be a potential solution to meeting the challenge and enhancing the sensor selectivity toward specific VFAs (Prévoteau and Rabaey, 2017). As proved in previous results, the specificity of the enriched bacterial community toward acetate did exist (Kaur et al., 2013, 2014; Kretzschmar et al., 2017). In addition to the above microbial methods, distillation or membrane separation of acetate from individual VFAs could be a possible solution. The linear fittings between the current density at specific reaction time (5 h) and initial concentration of each VFAs can be established with the correlation coefficient factors (*R*^2^) over 0.99, as shown in Supplementary Figure S2. This result indicates that the biosensor can also be used to measure propionate or butyrate each as a sole analyte.

### Effect of Sample Temperature on the Biosensor Performance

**FIGURE 5:**
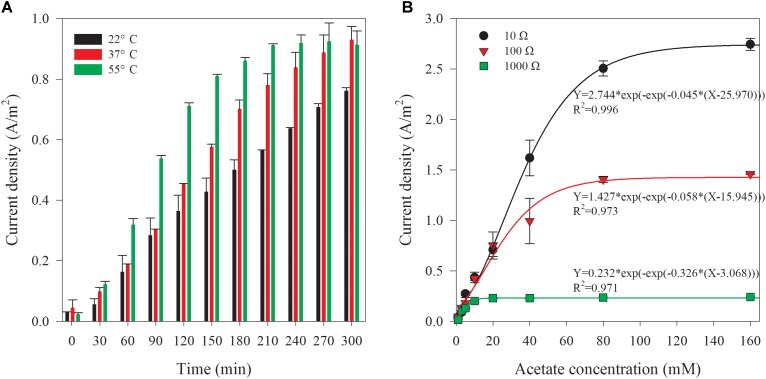
Current density variation along with time corresponding to the different initial temperature of sample **(A)** and the comparison of the current density from the biosensor with different external resistances at the reaction time of 5 h **(B)**.

Generally, the bacterial activities are susceptible to ambient temperature variations. Mass transfer and oxygen reduction rates catalyzed by Pt on the cathode are also affected by ambient temperature (Liu et al., 2005). The effect of sample temperature on the biosensor performance was thus investigated by filling the sample chamber with synthetic wastewater containing 20 mM acetate in different initial temperature. Initial temperatures of 37 and 55°C were chosen for simulating AD effluents from mesophilic and thermophilic conditions. Figure 5A shows the variation of current densities at different initial sample (artificial wastewater contains 20 mM acetate) temperatures in batch experiments. The current densities from the samples with an initial temperature of 37 and 55°C were 22.2 and 19.9% higher than that achieved at room temperature (22°C). The current density generated from the sample at 37°C showed a faster increase after 2-h reaction. Moreover at 55°C, 1-h reaction time was already enough to create a clearly higher current density. The time course of current density generated from the sample at 55°C showed the fastest increase during the period of 1 to 2 h, followed by an increase at a lower rate reaching a plateau after 4 h. Nevertheless, sample temperature of 37°C or 55°C can hardly affect the temperature of the cathode compartment, but may slightly increase the temperature in anode chamber which is adjacent to the sample chamber. Thus, the sample temperature cannot significantly affect the output of the measurement of the biosensor. However, as the current density can be affected by the temperature, attention should be taken to keep the temperature at a constant level.

### Effect of External Resistance on the Biosensor Performance

The external resistance determines the ratio between the voltage and the current for an MFC-type reactor (Aelterman et al., 2008). For a fixed MFC reactor, the current usually increases with the decrease of the external resistance; the opposite is also true for the voltage. Theoretically, maximum power generation and highest columbic efficiency could be achieved when the MFC is operated at the optimal external resistance that is equal to the internal resistance (Logan et al., 2006; Kiely et al., 2011). In this study, the performances of biosensor with 100 and 1000 Ω external resistance were also investigated. For this set of experiments, synthetic wastewaters containing various concentrations (1–160 mM) of acetate were used. At least, 1-week adaptation after a change of the external resistance was applied. Variation of current density of the biosensor with 100 or 1000 Ω external resistance was observed with the artificial AD effluents containing different acetate concentrations. The sigmoidal fit between current densities and initial acetate concentrations at varied reaction time (from 1 to 5 h) is shown in Supplementary Figures S3, S4, respectively. The current density increased along with the reaction time and initial acetate concentration in artificial wastewater. With 100 Ω external resistance, obvious slowdown of the current density increase was observed after around 3-h reaction time, when 80 or 160 mM acetate was dosed in the sample chamber. For the external resistance of 1000 Ω, the current density reached a platform after a period of increase when the acetate concentration surpassed 10 mM. The length of this period shortened with the increase of acetate concentration. The current density was not increase indicated that the saturated state of the biosensor was approached. Application of a 1000 Ω external resistance allows a relatively low initial acetate concentration (since 10 mM) or short reaction time (1.5 h for 160 mM acetate) that can reach the saturated state. The electron transfer rate from bacteria to anode and the electron flux through the external circuit are dependent on external resistance in MFCs (Jung and Regan, 2011). Therefore, the size of external resistance can be used for regulating the sensitivity of the biosensor. After the acetate accumulated to a certain level in anode chamber, the current generation of a MFC would limited by the external resistance rather than the substrate concentration (Jin et al., 2016). For a higher external resistance, the saturated substrate concentration would be lower (Catal et al., 2008).

A comparison of current densities obtained with different external resistances (10, 100, and 1000 Ω) at 5-h reaction time is shown in Figure 5B. The current densities of the biosensor with 1000 Ω external resistance almost stopped increasing when the acetate concentration in the artificial wastewater was over 10 mM. For the 100 Ω external resistance, the current densities kept increasing until the initial acetate concentration increased to 80 mM in the wastewater. However, the growth was slowed down when initial acetate concentration was higher than 20 mM, compared with that under the condition of 10 Ω external resistance. The results suggest that the detection range of the biosensor with a 5-h reaction can be lower when a higher external resistance is adopted. For the purpose of AD process monitoring, a lower external resistance (10 and 100 Ω) is recommended.

### Verification of the Biosensor With AD Effluent

**FIGURE 6:**
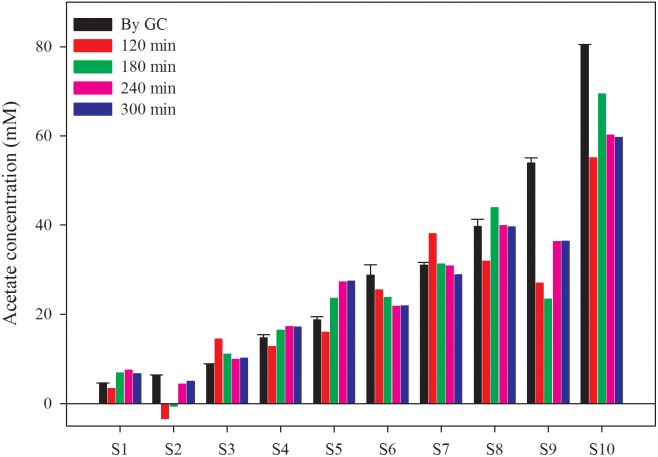
Acetate concentration measured by the biosensor and GC. The sample was 5 times diluted AD effluent from a CSTR reactor fed with cow manure mixed with different acetate concentration.

To verify the applicability of this biosensor in AD process monitoring, the AD effluent from a 10 L CSTR fed with cow manure was tested. The characteristics of the AD effluent are shown in Table 1. The AD effluent was five times diluted and amended with various concentration of acetate before measurement. In addition, the acetate concentration in all samples was also measured by GC, which served as the reference value in this study. Supplementary Figure S5 shows the current density time courses of these samples. The acetate concentration measured by the biosensor was calculated based on Equation 2 and the current densities obtained at specific reaction time (2, 3, 4, and 5 h), as shown in Figure 6. The average relative differences between the results from GC measurement and the biosensor at different reaction time (2, 3, 4, and 5 h) were 40.2, 32.6, 26.1, and 24.2%, respectively. It implies that a longer reaction time can benefit the stability of the biosensor. However, it is still hard to say the results from the biosensor were close to the reference values, further efforts in increasing the accuracy are needed. Beside, in order to enhance the stability and avoid the adverse effect of suspended solid, an automatic pre-treatment process such as filtration could be developed to fully address the issue of membrane clog. Although the correspondence of the measurements still need improvement, the biosensor has potential also considering its simplicity, as an alternative way for AD process monitoring.

**Table 1:** Characteristics of the anaerobic digested slurry.

Parameter	Data	Parameter	Data	Parameter	Data
TS (%)	2.072 ± 0.003	Acetic acid (mM/L)	4.63 ± 0.18	Isovaleric acid (mM/L)	0.10 ± 0.00
VS (%)	1.262 ± 0.008	Propionic acid (mM/L)	0.25 ± 0.01	Valeric acid (mM/L)	<0.1
pH	8.54	Isobutyric acid (mM/L)	<0.1	Hexanoic acid (mM/L)	<0.1
EC (mS/cm)	17.22	Butyric acid (mM/L)	0.49 ± 0.08	NH_4_^+^ (mM/L)	152.81 ± 0.99

### Perspectives

The conventional methods for VFAs monitoring in AD process mainly use the gas or liquid chromatography and titration. The expensive equipment and dedicated sample preparation are indispensable for VFAs analysis by chromatography. Removing the solids in the sample is necessary to obtain an accuracy measurement with titration which can only detect the total VFAs (Purser et al., 2014). The accuracy and the reaction time of the current biosensor still need to be improved to replace these methods, but it maximally simplified the sample preparation to obtain a reasonable accuracy results. Moreover, it is promising with high potential in improving the accuracy and shortening the reaction time, most importantly achieving on-line measurement which is essential for the AD process in future.

In comparison with other bioelectrochemical biosensors for VFAs measurement, the present biosensor has the following main advantages. Firstly, the detection range was large enough to satisfy the regiments of acetate detection in AD process with acceptable accuracy with 3 to 5-h reaction. Secondly, the sample was dosed in the sample chamber that was physically separated by AEM with the anode chamber, which could maximize the stability of the biosensor. Thirdly, EC and pH of the medium in anode and cathode chamber only slightly changed after batch experiment for 5 h (Table 2). Fourthly, the current through the external circuit instead of the complex electrochemical was monitored as a sensing signal. Fifthly, the biosensor worked under conditions of no sterility, no agitation and room temperature, which simplified its practical applications to a large extent. With respect to selectivity, the challenge of developing a biosensor with high specificity toward acetate is not fully addressed. Nevertheless, the application of the biosensor could still be promising and serve as a simple tool for AD process monitoring.

**Table 2:** Variation of pH and EC during the batch experiments.

Parameter	Before batch			After batch		
	Sample	Anode	Cathode	Sample	Anode	Cathode
pH	7.55 ± 0.75	7.31 ± 0.05	7.33 ± 0.06	7.51 ± 0.55	7.28 ± 0.08	7.34 ± 0.07
EC (mS/cm)	15.75 ± 4.76	6.64 ± 0.04	6.64 ± 0.04	15.48 ± 4.76	6.50 ± 0.13	6.67 ± 0.15
T (°C)	21.49 ± 1.26	21.85 ± 0.90	21.43 ± 1.18	22.63 ± 0.83	22.51 ± 0.82	22.44 ± 0.82

*Before batch: n = 65. After batch: n = 103.*

## Conclusion

The present work investigated the feasibility of a three-chamber bioelectrochemical sensor for acetate detection during AD process. Sigmoidal relationship between the current densities and acetate concentrations was up to 160 mM in artificial wastewater at the specific reaction time. The average relative standard deviation of the current densities obtained at various acetate concentrations was below 10% for a reaction time of 4 or 5 h. The mechanism of the substrates transportation to the anode chamber in this biosensor was concentration diffusion. Other VFAs would also transfer through the AEM and be utilized in the anode for the current generation. Especially, current densities, generated from the propionate as the sole analyte, could reach the same level compared with that from the acetate under the same concentration. High temperature (37 and 55°C) samples could boost the current density, but lower the temperature before the analysis, which was necessary to obtain accurate results. High external resistance could lower the saturated concentration of substrate, and shorten the detection range of the biosensor. The application of a lower external resistance (10 and 100 Ω) is recommended to satisfy the measurement requirements of the acetate monitoring during the AD process. The proposed biosensor is promising as a simple approach for the AD process monitoring.

## Author Contributions

YZ and HS conceived the study, designed the experiments, and participated in drafting the article. IA, SW, and RD revised it critically for important intellectual content. All authors approved the manuscript submission.

## Conflict of Interest Statement

The authors declare that the research was conducted in the absence of any commercial or financial relationships that could be construed as a potential conflict of interest.
