# Supplementary material for: The Potential of Bioelectrochemical Sensor for Monitoring of Acetate During Anaerobic Digestion: Focusing on Novel Reactor Design
Source: Front Microbiol. 2019 Jan 15;9:3357. doi: 10.3389/fmicb.2018.03357 (PMC6340975; doi:10.3389/fmicb.2018.03357)
Supplement: Supplementary file 1 [file Data_Sheet_1.pdf]

Supplementary Material

The Potential of Bioelectrochemical Sensor for Monitoring of Acetate During Anaerobic Digestion: Focusing on Novel Reactor Design

Hao Sun, Irini Angelidaki, Shubiao Wu*, Renjie Dong, Yifeng Zhang*

*** Correspondence:** Dr. Yifeng Zhang: yifz@env.dtu.dk; yifzmfc@gmail.com

Dr. Shubiao Wu: wushubiao@gmail.com

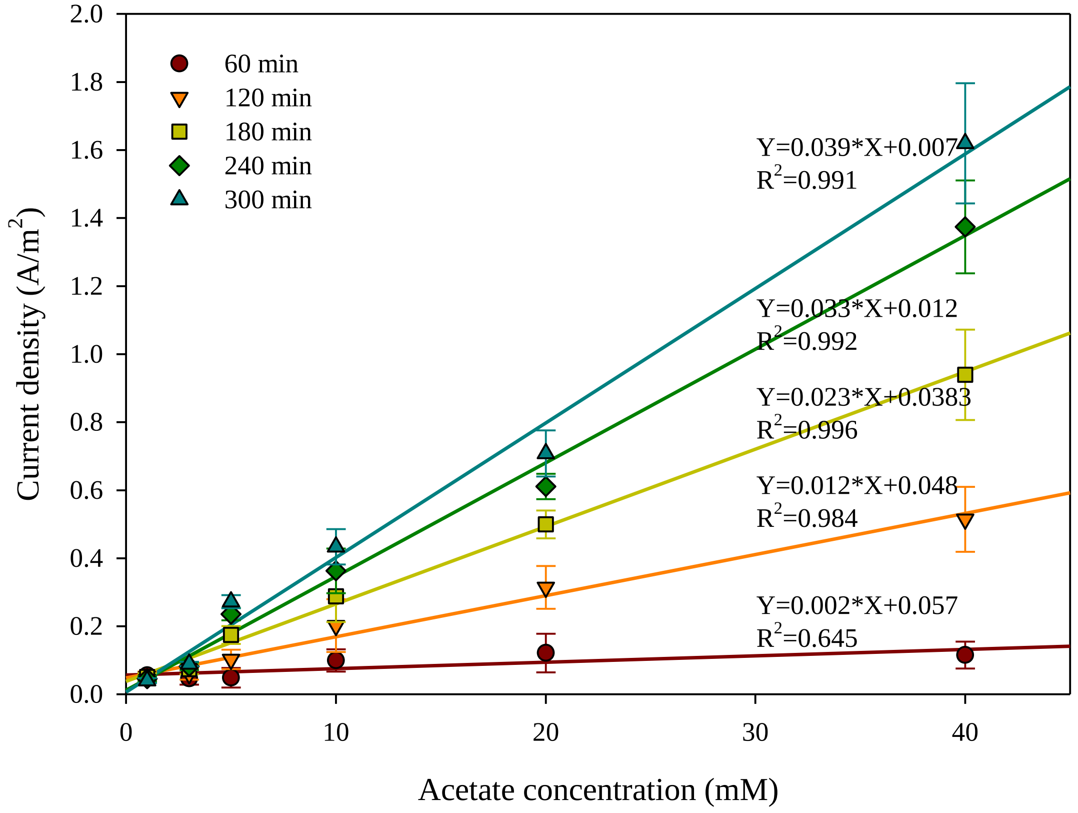

Supplementary Figure 1. Linear fitting of current densities and acetate concentrations (1-40 mM) in the artificial AD effluent at varied reaction time (from 1 to 5 hour).

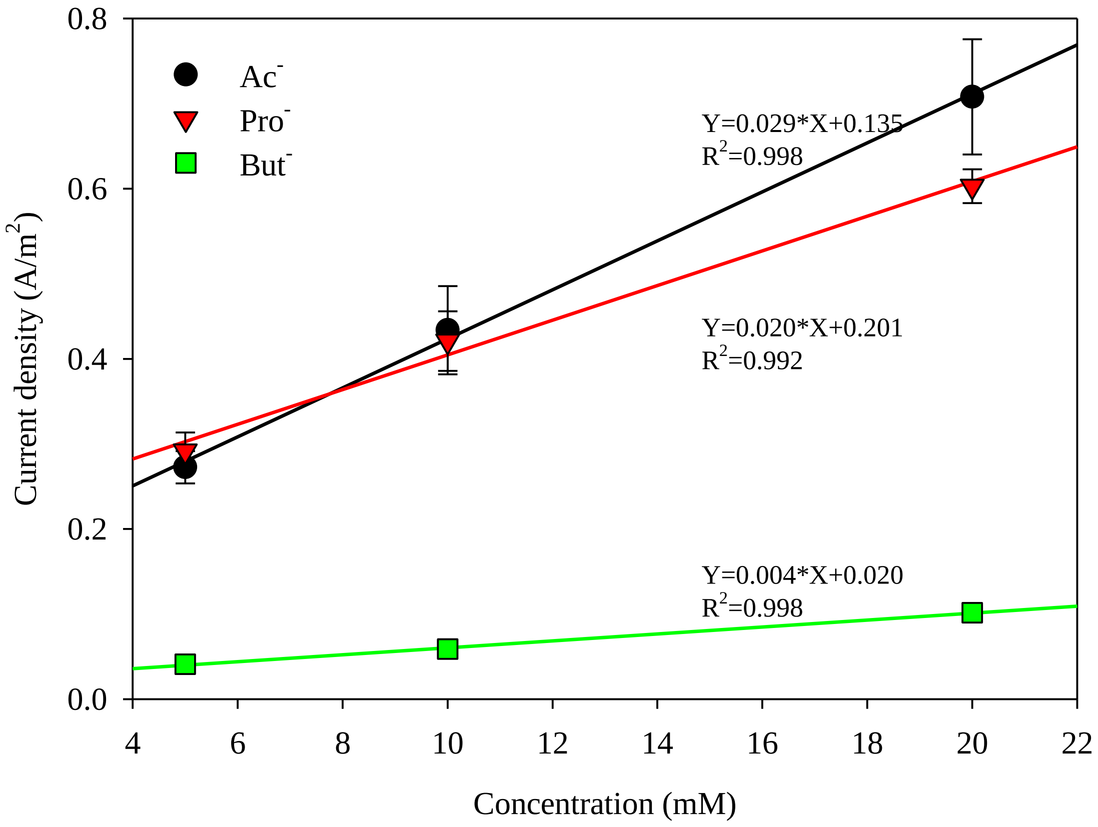

Supplementary Figure 2. Linear fitting between the current densities of the bioreactor after 5 hours reaction corresponding to different VFAs in the artificial AD effluent.

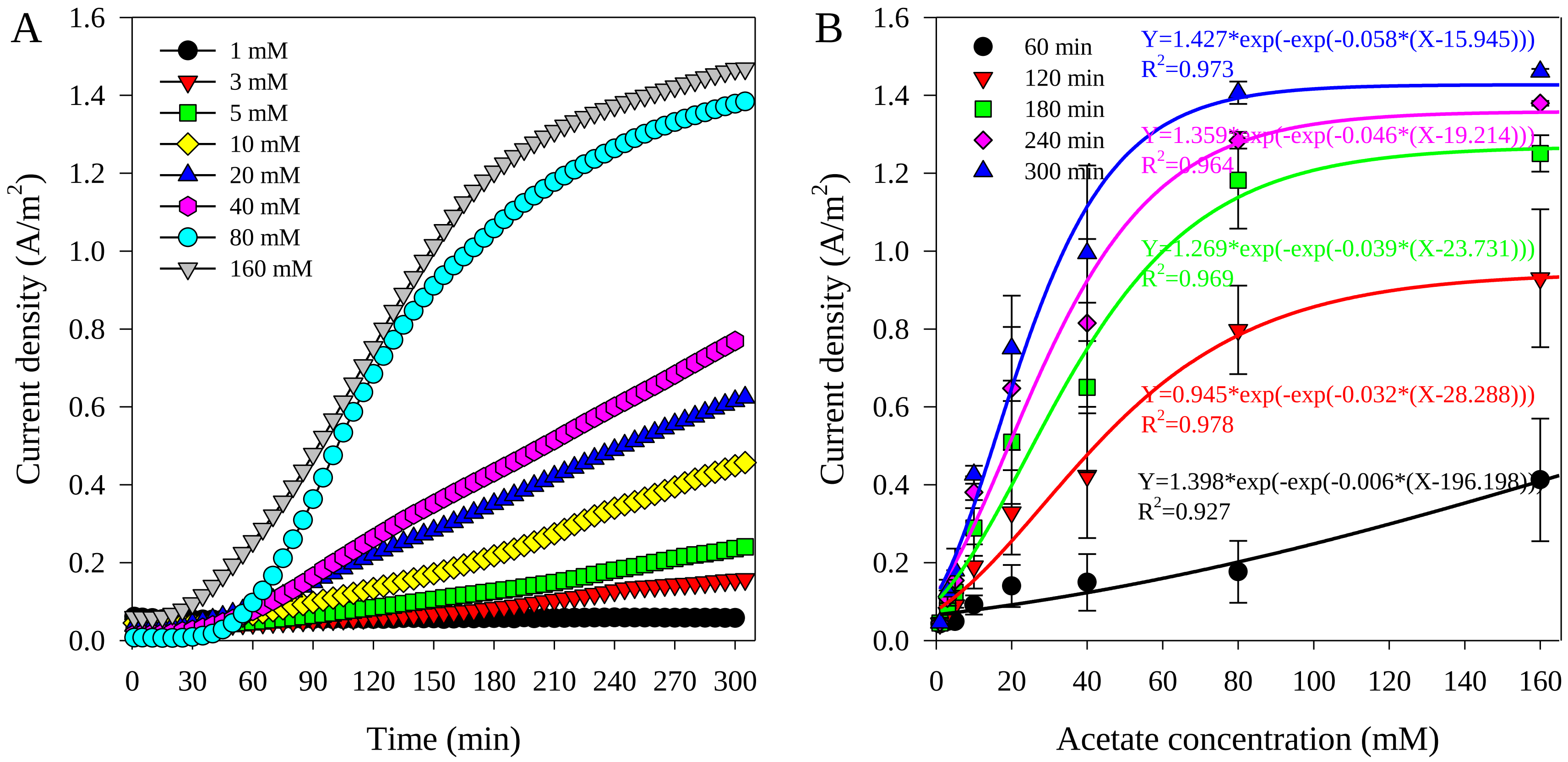

Supplementary Figure 3. Typical variation of current density of the biosensor with 100 Ω external resistance corresponding to different acetate concentration in the artificial AD effluent (A) and the relationship between current densities and acetate concentrations at varied reaction time (from 1 to 5 hour) (B)

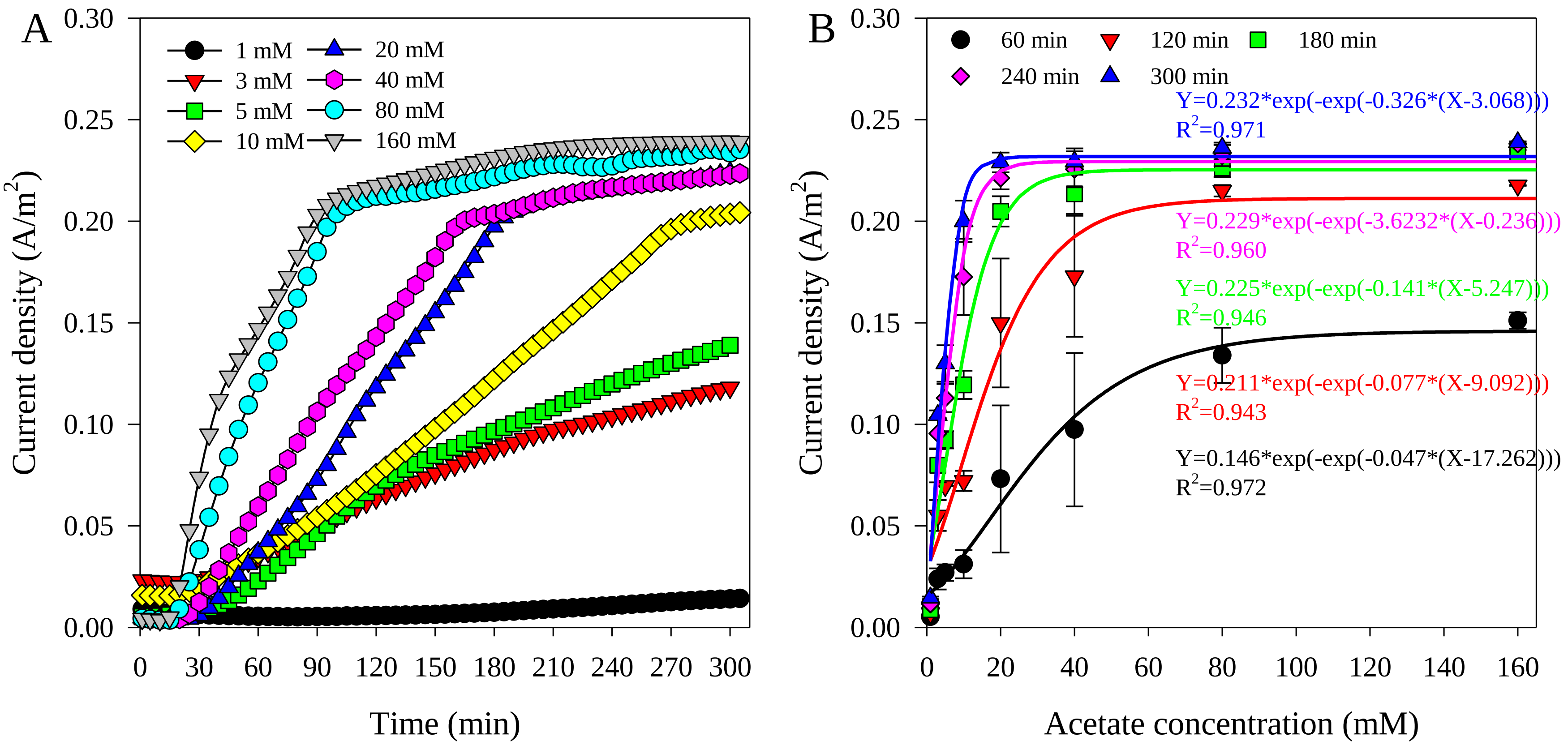

Supplementary Figure 4. Typical variation of current density of the biosensor with 1000 Ω external resistance corresponding to different acetate concentration in the artificial AD effluent (A) and the relationship between current densities and acetate concentrations at varied reaction time (from 1 to 5 hour) (B)

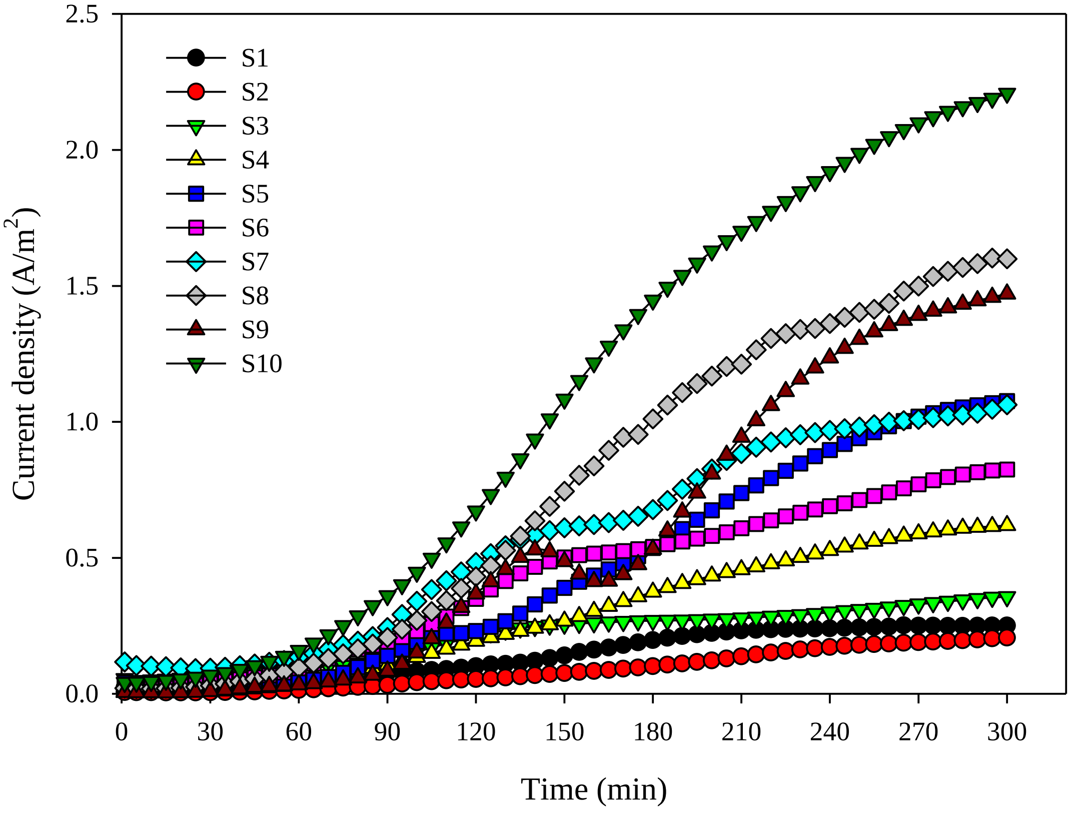

Supplementary Figure 5. Current density time courses of samples that were 5 times dilution of AD effluent from a CSTR reactor fed with cow manure mixed with different concentrations of acetate.
